# Supplementary material for: Graphene Bridge for Photocatalytic Hydrogen Evolution with Gold Nanocluster Co-Catalysts
Source: Nanomaterials (Basel). 2022 Oct 17;12(20):3638. doi: 10.3390/nano12203638 (PMC9612079; doi:10.3390/nano12203638)
Supplement: Supplementary file 1 [file nanomaterials-12-03638-s001.zip › nanomaterials-1950380-supplementary.pdf]

Supplementary Materials

Graphene Bridge for Photocatalytic Hydrogen Evolution with Gold Nanocluster Co-Catalysts

Hanieh Mousavi [1], Thomas D. Small [1], Shailendra K. Sharma [2], Vladimir B. Golovko [2], Cameron J. Shearer [1,*] and Gregory F. Metha [1,*]

[1] Department of Chemistry, University of Adelaide, Adelaide SA 5005, Australia;
[2] The MacDiarmid Institute for Advanced Materials and Nanotechnology, School of Physical and Chemical Sciences, University of Canterbury, Christchurch 8140, New Zealand;
* Correspondence: cameron.shearer@adelaide.edu.au (C.J.S.); greg.metha@adelaide.edu.au (G.F.M.)

Contents

1. List of Figures 3

2. Supplementary experimental details 4

2.1 Methodology optimisation of photocatalysts preparation 4

2.2.1 The effect of Au mass loading on HER activity of $Au_{101}NC$-$AlSrTiO_3$:rGO 4

2.2.2 The effect of heating in air and under vacuum on HER activity of $Au_{101}NC$-$AlSrTiO_3$:rGO 4

3. Supplementary experimental results and discussion 4

3.1 Physical characterization 4

3.2 Photocatalytic hydrogen evolution activity 7

3.2.1 The effect of Au mass loading on HER activity of $Au_{101}NC$-$AlSrTiO_3$:rGO 7

3.2.2 The effect of heating in air and under vacuum on HER activity of $Au_{101}NC$-$AlSrTiO_3$:rGO 8

3.2.3 Appearance of $Au_{101}NC$-$AlSrTiO_3$:rGO and $Au_{101}NC$-rGO:$AlSrTiO_3$ before and after photocatalytic HER ... 8

4. References 10

1. List of Figures and Tables

Figure S1. The SEM image of $AlSrTiO_3$. 4

Figure S2. (a-b) HAADF-STEM images and (c) size distribution histogram of unsupported Au_{101}NC dropcast onto a TEM grid from methanol solution. 5

Figure S3. BF-STEM images of Au_{101}NC-$AlSrTiO_3$:rGO (a) before and (b) after HER and Au_{101}NC-rGO:$AlSrTiO_3$ (c) before and (d) after HER. Au mass loading: 0.05 wt%. Annealing was performed at 210 °C in air for 15 min. 5

Figure S4. EDX spectra of a chosen spot on (a) rGO in Au_{101}NC-$AlSrTiO_3$:rGO and (b) $AlSrTiO_3$ in Au_{101}NC-rGO:$AlSrTiO_3$. Annealing was performed at 210 °C in air for 15 min. The inserts are the HAADF-STEM images of the chosen area. 6

Figure S5. EDX spectra of a chosen spot on rGO in (a) Au_{101}NC-$AlSrTiO_3$:rGO-HER and (b) Au_{101}NC-rGO:$AlSrTiO_3$-HER. Annealing was performed at 210 °C in air for 15 min. The inserts are the HAADF-STEM images of the chosen area. 6

Figure S6. UV Vis DRS of Au_{101}NC-$AlSrTiO_3$:rGO and Au_{101}NC-rGO:$AlSrTiO_3$ compared to $AlSrTiO_3$ (a) before and (b) after HER. Annealing was performed in air at 210 °C for 15 min. 7

Figure S7. Sacrificial liquid phase H_2 photocatalysis of Au_{101}NC-$AlSrTiO_3$:rGO (210 °C in air for 15 min) with Au mass loading of 0.05 and 1 wt%.(Conditions: 1:2 Methanol:Water, 365 nm at 83 mW/cm^2, 2 h reaction time). 8

Figure S8. Sacrificial liquid phase H_2 photocatalysis of unannealed, annealed (air), and annealed (vacuum) Au_{101}NC-$AlSrTiO_3$:rGO. Au mass loading 1 wt%, 1:2 Methanol:Water, LED 365 nm at 83 mW/cm^2 for 2 h. Annealing was performed at 210 °C for 15 min. 8

Figure S9. Photographs showing sample dispersions of (a) Au_{101}NC-$AlSrTiO_3$:rGO and (b) Au_{101}NC-rGO:$AlSrTiO_3$ before (left images) and after (right images) photocatalytic HER. Au mass loading 0.05 wt%, 1:2 Methanol:Water, LED 365 nm at 83 mW/cm^2 for 2h. Annealing was performed at 210 °C in air for 15 min. 9

Figure S10. Photographs showing dispersions of Au_{101}NCs (1 mg mL^{-1}) in (a) 1:2 Methanol:Water and (b) Methanol. 10

Table S1. Average external quantum efficiencies (EQE)% for synthesized photocatalysts, assuming 2 electrons per H_2 molecule produced.

2. Supplementary experimental details

2.1 Methodology optimisation of photocatalysts preparation

2.2.1 The effect of Au mass loading on HER activity of $Au_{101}NC-AlSrTiO_3$:rGO

To make 10 mg $Au_{101}NC-AlSrTiO_3$:rGO with 0.05 and 1 wt% Au, 6.4 µL and 128 µL of an $Au_{101}NC$ dispersion (1 mg mL^{-1} ligated Au mass) were added dropwise to 10 mg of $AlSrTiO_3$ dispersed in 2 mL methanol in a porcelain evaporating dish and homogenised using bath sonication at RT until the solvent completely evaporated. The as-obtained $Au_{101}NC-AlSrTiO_3$ samples were dispersed and homogenised in 2 mL methanol via bath sonication (2 min). Then 16.6 µL of the as-synthesized rGO dispersion was added dropwise to each dispersion with bath sonication at RT until the solvent completely evaporated. The as-obtained $Au_{101}NC-AlSrTiO_3$:rGO samples were annealed in a muffle furnace at 210 °C in air for 15 min.

2.2.2 The effect of heating in air and under vacuum on HER activity of $Au_{101}NC-AlSrTiO_3$:rGO

The as-obtained $Au_{101}NC-AlSrTiO_3$:rGO with 1 wt% of Au was placed in a porcelain evaporating dish was annealed in air in a muffle furnace and vacuum (< 0.01 bar) in a tube furnace (Across International, STF1200, quartz tube) at 210 °C for 15 min. This sample was compared with unannealed $Au_{101}NC-AlSrTiO_3$:rGO using photocatalysis.

3. Supplementary experimental results and discussion

3.1 Physical characterization

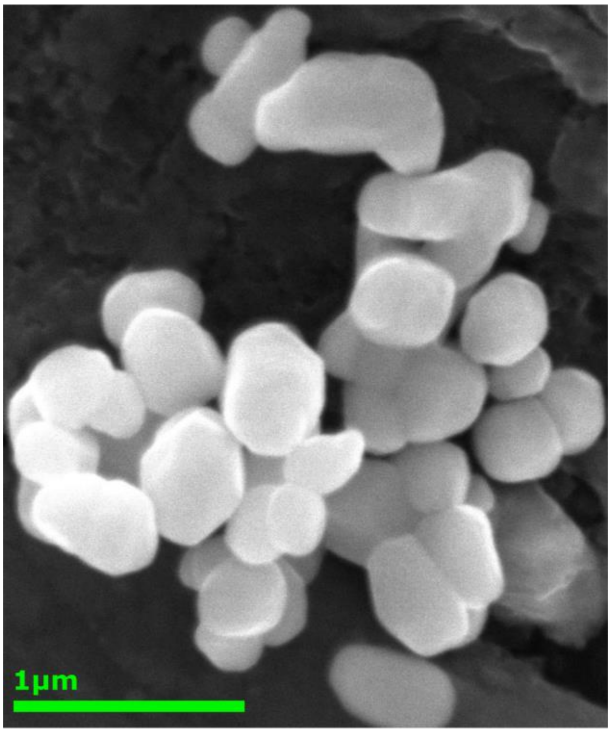

Figure S1. SEM image of $AlSrTiO_3$.

In order to determine if the size of the AuNCs changes during the formation of the photocatalyst, it is first required to benchmark the size of the as-prepared AuNCs. Figure S2(a-c) presents the high-resolution HAADF-STEM images and the measured cluster size histogram fitted to a log-normal distribution. The $Au_{101}NCs$ have a mean diameter of 1.74 ± 0.29 nm and a mode of 1.52 nm. 3.9% of the clusters have diameters smaller than 1 nm and 25.6 % larger than 2 nm. In our previous publication which used a different batch of $Au_{101}NC$, we measured an average diameter of 1.3 ± 0.4 nm with 26.4% of particles less than 1 nm and 3.4 % greater than 2 nm [1].

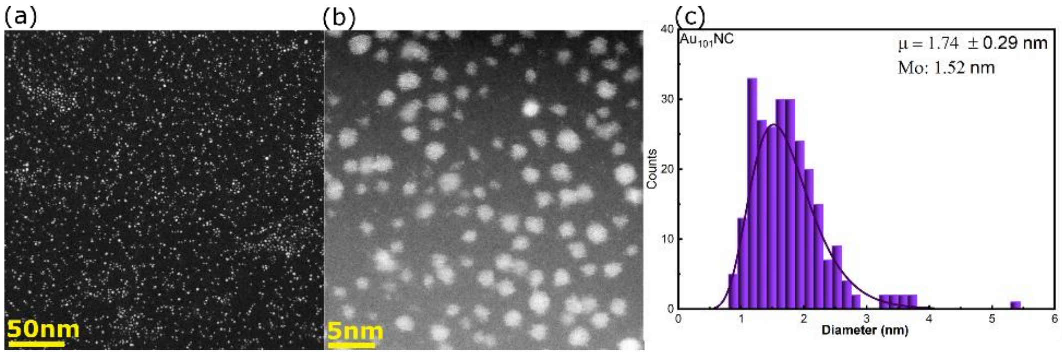

Figure S2. (a-b) HAADF-STEM images and (c) size distribution histogram of unsupported Au_{101}NC dropcast onto a TEM grid from methanol solution.

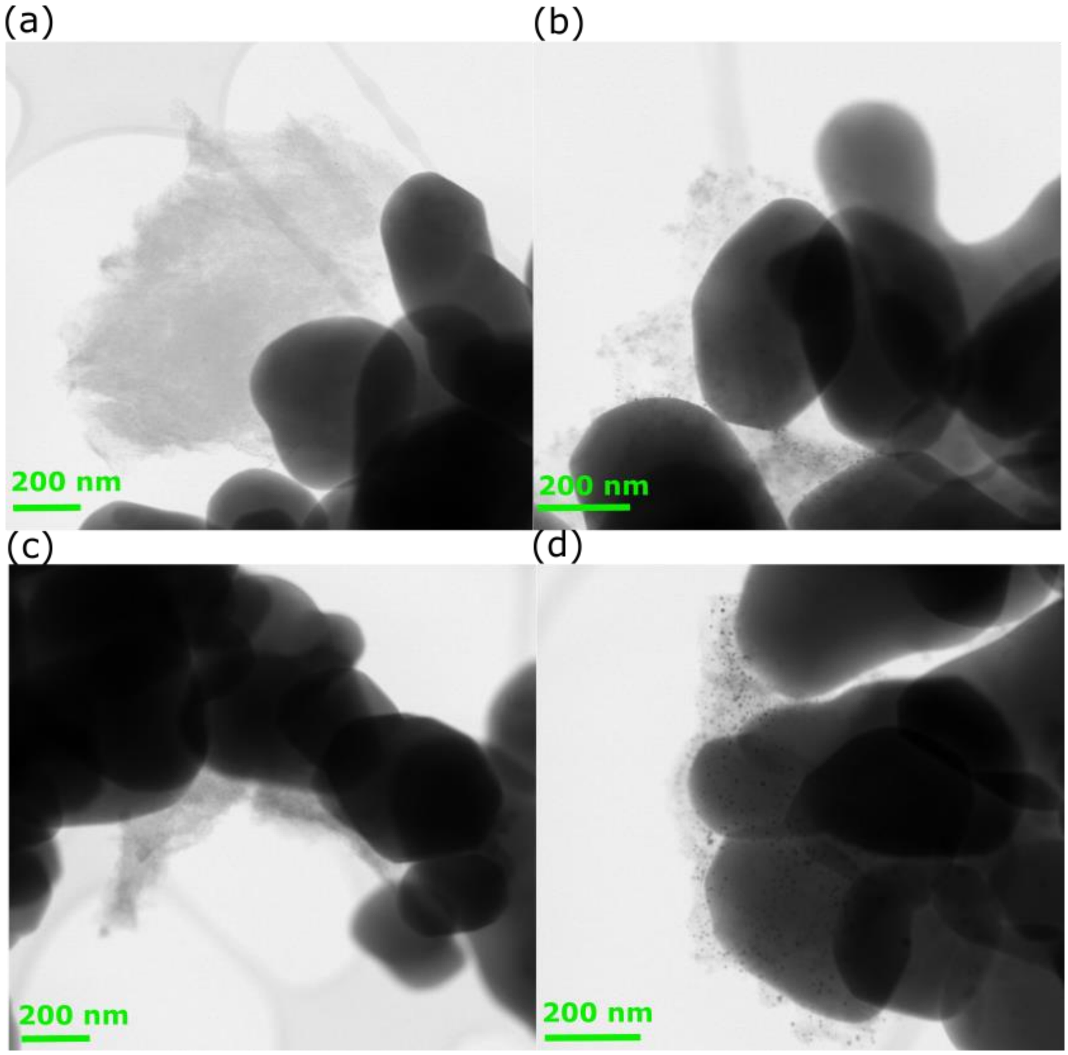

Figure S3. BF-STEM images of Au_{101}NC-$AlSrTiO_3$:rGO (a) before and (b) after HER and Au_{101}NC-rGO:$AlSrTiO_3$ (c) before and (d) after HER. Au mass loading: 0.05 wt%. Annealing was performed at 210 °C in air for 15 min.

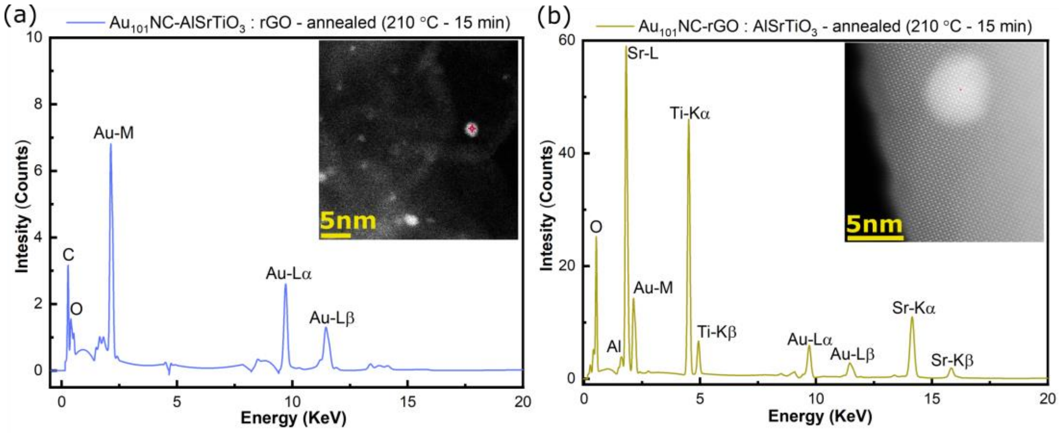

Figure S4. EDX spectra of the selected spot on (a) rGO in Au_{101}NC-$AlSrTiO_3$:rGO and (b) $AlSrTiO_3$ in Au_{101}NC-rGO:$AlSrTiO_3$. Annealing was performed at 210 °C in air for 15 min. The inserts are the HAADF-STEM images of the chosen area.

EDX spectra from a selected area of rGO from Au_{101}NC-$AlSrTiO_3$:rGO-HER and Au_{101}NC-rGO:$AlSrTiO_3$-HER samples is shown in Figure S5(a-b). Interestingly, in both samples, signal from $SrTiO_3$ is seen on rGO after, but not before, HER. Future work is required to find out how this occurs and its possible effect on photocatalytic activity.

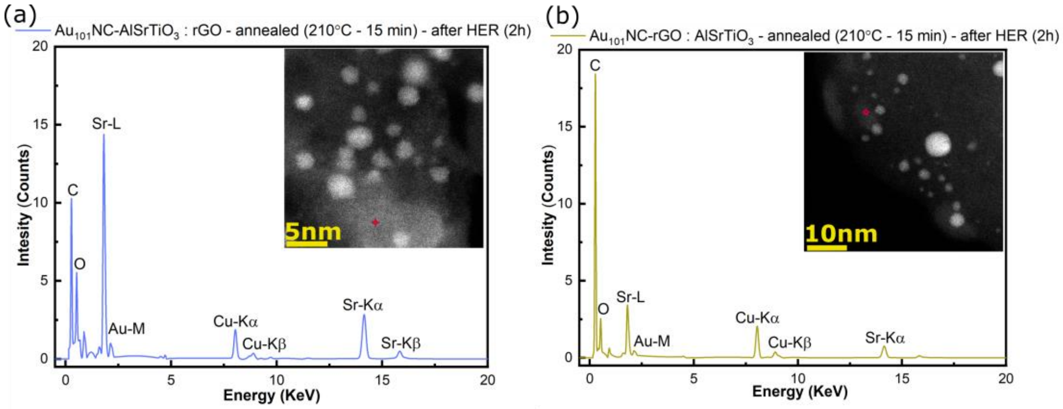

Figure S5. EDX spectra of a selected spot on rGO in (a) Au_{101}NC-$AlSrTiO_3$:rGO-HER and (b) Au_{101}NC-rGO:$AlSrTiO_3$-HER. Annealing was performed at 210 °C in air for 15 min. The inserts are the HAADF-STEM images of the chosen area.

UV-Vis DRS was used to measure the optical properties and agglomeration state of Au_{101}NC for the as-prepared Au_{101}NC-$AlSrTiO_3$:rGO and Au_{101}NC-rGO:$AlSrTiO_3$ before and after photocatalysis and compared to $AlSrTiO_3$ (Figure S6(a-b)). In Figure S6(a), $AlSrTiO_3$ exhibits a typical absorption edge at 387 nm indicating a band gap of 3.2 eV. The bandgap of $AlSrTiO_3$ and the UV light absorption intensity are effectively identical upon addition of both rGO and Au_{101}NC onto $AlSrTiO_3$, indicating that the electronic structure and optical properties of the $AlSrTiO_3$ are not altered. The slight absorption from 385 – 800 nm is due to the visible light absorbance of rGO [2]. The photocatalysis was performed at 365 nm (3.4 eV), where $AlSrTiO_3$ has strong absorbance and Au_{101}NC and rGO has negligible UV light absorbance. Additionally, no peak at ca. 520 nm due to Au surface plasmon resonance was observed, indicating that there is no agglomeration of Au_{101}NC deposited onto either rGO or $AlSrTiO_3$. Likewise, evolving hydrogen under illumination did not cause agglomeration of the Au_{101}NC, as no peak at 520 nm was observed for samples that were measured after performing HER for two hours.

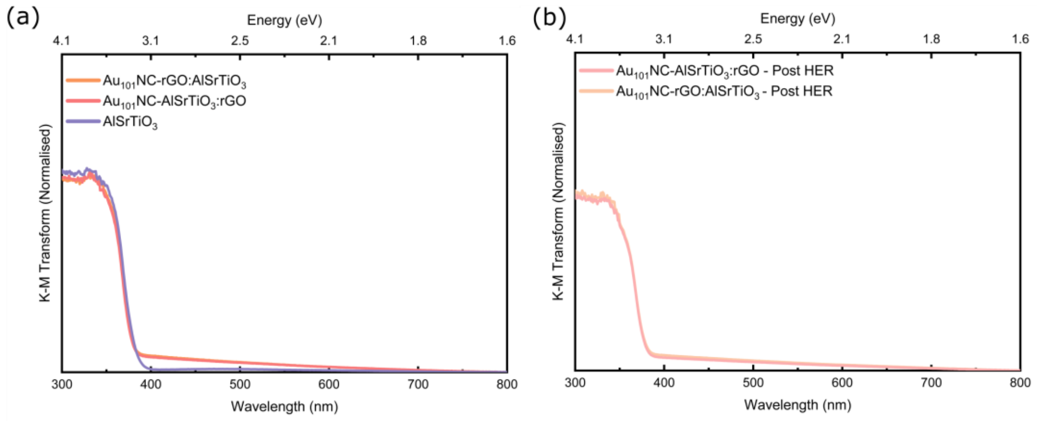

Figure S6. UV-Vis DRS of Au_{101}NC-$AlSrTiO_3$:rGO and Au_{101}NC-rGO:$AlSrTiO_3$ compared to $AlSrTiO_3$ (a) before and (b) after HER. Annealing was performed in air at 210 °C for 15 min.

3.2 Photocatalytic hydrogen evolution activity

Table S1. Average external quantum efficiencies (EQE)% for synthesized photocatalysts, assuming 2 electrons per H_2 molecule produced.

**Photocatalyst**	**Average H_2 EQE% (at 2 hours)**
rGO	0.00046
$AlSrTiO_3$	0.00079
Au_{101}NC-rGO	0.00035
Au_{101}NC-rGO:$AlSrTiO_3$	0.04304
Au_{101}NC-$AlSrTiO_3$	0.00513
Au_{101}NC-$AlSrTiO_3$:rGO	0.04964
rGO-$AlSrTiO_3$	0.00507

3.2.1 The effect of Au mass loading on HER activity of Au_{101}NC-$AlSrTiO_3$:rGO

Figure S7 presents the effect of Au mass loading (0.05% and 1 wt%) on the photocatalytic HER activity in the Au_{101}NC-$AlSrTiO_3$:rGO nanocomposite. Increasing the Au mass loading by 20 times results in a × 1.6 increase in photocatalytic activity with the average H_2 production rate changing from 334 ± 24 to 539 ± 14 nmol h^{-1}.

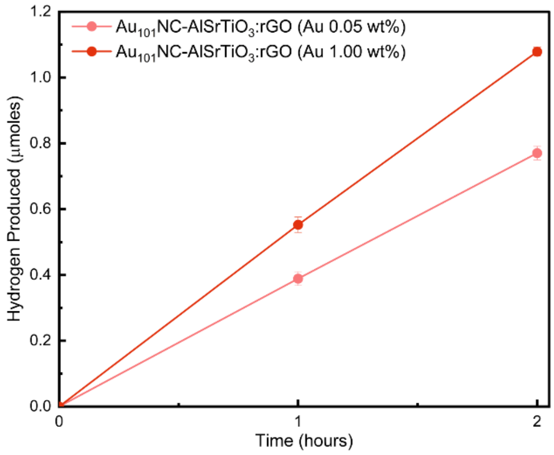

Figure S7. Sacrificial liquid phase H_2 photocatalysis of Au_{101}NC-$AlSrTiO_3$:rGO (210 °C in air for 15 min) with Au mass loading of 0.05 and 1 wt%.(Conditions: 1:2 Methanol:Water, 365 nm at 83 mW/cm^2, 2 h reaction time).

3.2.2 The effect of heating in air and under vacuum on HER activity of Au_{101}NC-$AlSrTiO_3$:rGO

To optimize the synthesis of photocatalysts, different annealing methods (air and vacuum) were applied. Figure S8 presents the effect of heating in air and under vacuum at 210 °C for 15 min on the photocatalytic HER activity of Au_{101}NC-$AlSrTiO_3$:rGO with 1% Au mass loading compared with unannealed samples. The photocatalytic activity was found to increase in the order; air annealing > vacuum annealing > not annealed, with average H_2 production rates of 1110, 510, and 320 nmol after 2h, respectively. Future work is required to find out the reasons for high HER activity of photocatalysts in air than vacuum.

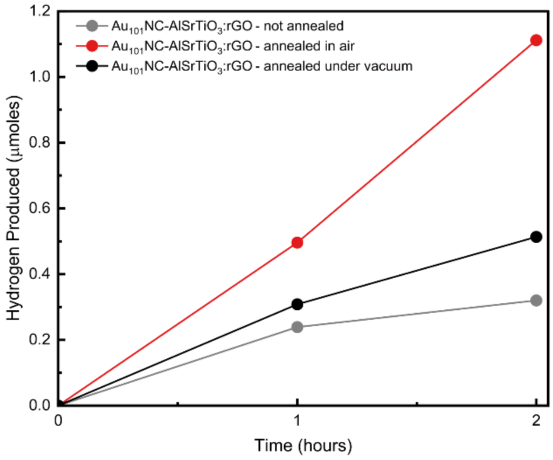

Figure S8. Sacrificial liquid phase H_2 photocatalysis of unannealed, annealed (air), and annealed (vacuum) Au_{101}NC-$AlSrTiO_3$:rGO. Au mass loading 1 wt%, 1:2 Methanol:Water, LED 365 nm at 83 mW/cm^2 for 2 h. Annealing was performed at 210 °C for 15 min.

3.2.3 Appearance of Au_{101}NC-$AlSrTiO_3$:rGO and Au_{101}NC-rGO:$AlSrTiO_3$ before and after photocatalytic HER

Figure S9(a-b) shows photographs of the $AlSrTiO_3$:rGO and Au_{101}NC-rGO:$AlSrTiO_3$ dispersion solutions used for photocatalytic HER. There is no observable change in the solution colour indicating the resistance of the as-prepared nanocomposites toward agglomeration (i.e. no presence of Au surface plasmon resonance), consistent with the DRS.

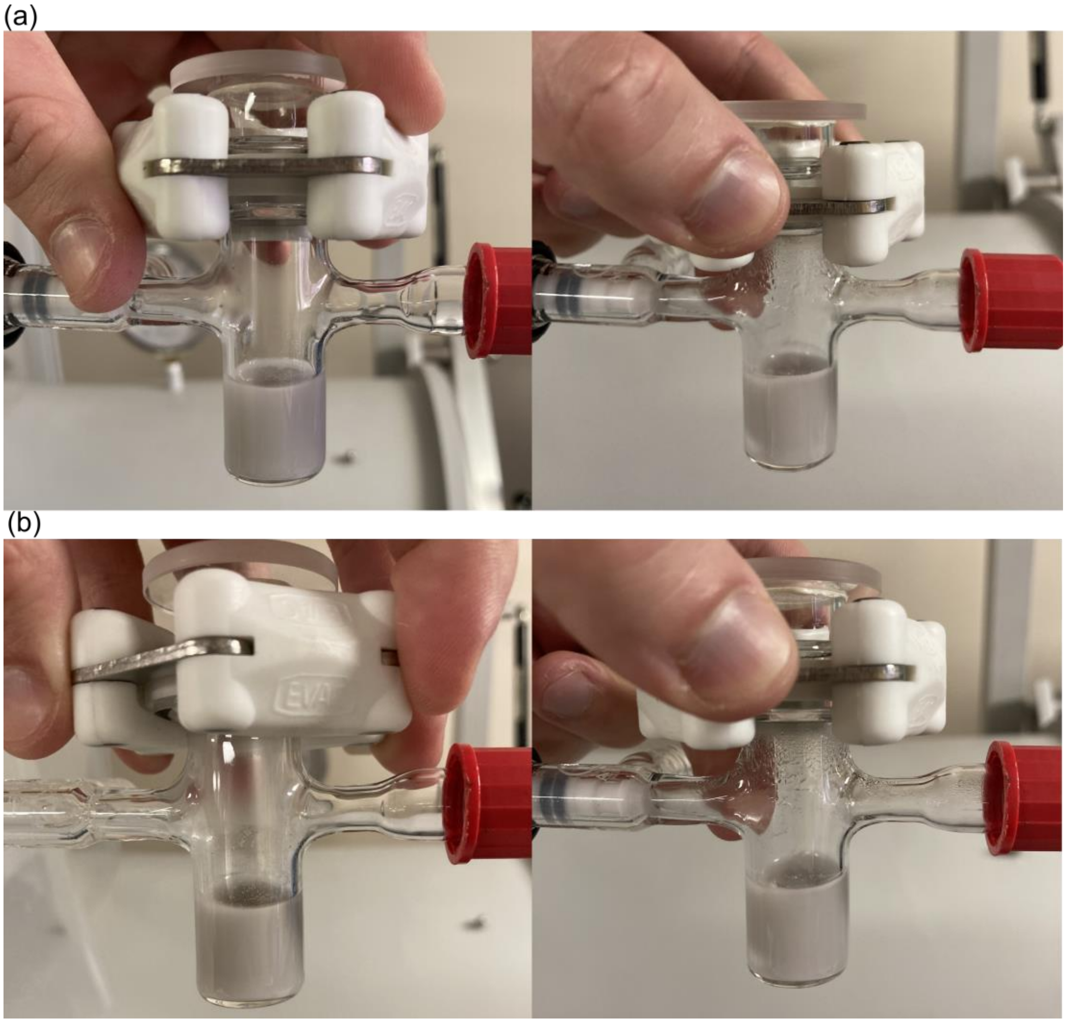

Figure S9. Photographs showing sample dispersions of the (a) Au_{101}NC-Al$SrTiO_3$:rGO and (b) Au_{101}NC-rGO:Al$SrTiO_3$ samples before (left images) and after (right images) photocatalytic HER. Au mass loading 0.05 wt%, 1:2 Methanol:Water, LED 365 nm at 83 mW/cm^2 for 2h. Annealing was performed at 210 °C in air for 15 min.

Figure S10(a-b) shows the solubility of Au_{101}NC (1 mg mL^{-1}) in (a) 1:2 Methanol:Water and (b) Methanol. Au_{101}NC is highly soluble in methanol while it has very limited solubility in 1:2 Methanol:Water, indicating the adsorbed Au_{101}NC is unlikely to desorb from the surface of Al$SrTiO_3$/rGO in 1:2 Methanol:Water during photocatalysis.

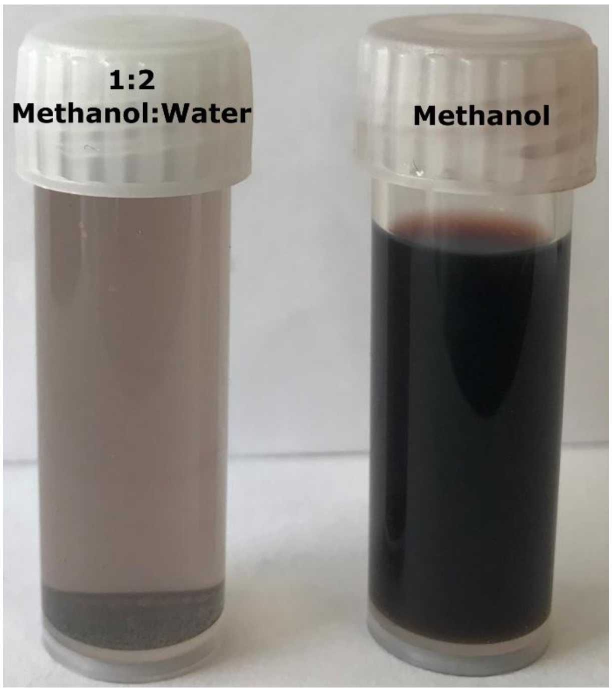

Figure S10. Photographs showing dispersions of Au_{101}NC (1 mg mL^{-1}) in (a) 1:2 Methanol:Water and (b) Methanol.

4. References

1. Mousavi, H.; Yin, Y.; Howard-Fabretto, L.; Sharma, S.K.; Golovko, V.; Andersson, G.G.; Shearer, C.J.; Metha, G.F. Au101–rGO nanocomposite: Immobilization of phosphine-protected gold nanoclusters on reduced graphene oxide without aggregation. *Nanoscale Adv* **2021**, *3*, 1422-1430.
2. Albero, J.; Mateo, D.; García, H.J.M. Graphene-based materials as efficient photocatalysts for water splitting. *Molecules* **2019**, *24*, 906.
